# Influence of COMT val158met Genotype on the Depressed Brain during Emotional Processing and Working Memory

**DOI:** 10.1371/journal.pone.0073290

**Published:** 2013-09-12

**Authors:** Esther M. Opmeer, Rudie Kortekaas, Marie-José van Tol, Nic J. A. van der Wee, Saskia Woudstra, Mark A. van Buchem, Brenda W. Penninx, Dick J. Veltman, André Aleman

**Affiliations:** 1 Neuroimaging Center, Department of Neuroscience, University Medical Center Groningen and University of Groningen, Groningen, The Netherlands; 2 Department of Psychiatry, Leiden University Medical Center, Leiden University, Leiden, The Netherlands; 3 Leiden Institute for Brain and Cognition, Leiden University, Leiden, The Netherlands; 4 Department of Psychiatry, VU University Medical Center, Amsterdam, The Netherlands; 5 Department of Medical Genomics, VU University Medical Center, Amsterdam, The Netherlands; 6 Neuroscience Campus Amsterdam, VU University, Amsterdam, The Netherlands; 7 Department of Radiology, Leiden University Medical Center, Leiden, The Netherlands; 8 Department of Psychology, University of Groningen, Groningen, The Netherlands; University of Wuerzburg, Germany

## Abstract

Major depressive disorder (MDD) has been associated with abnormal prefrontal-limbic interactions and altered catecholaminergic neurotransmission. The val158met polymorphism on the catechol-O-methyltransferase (COMT) gene has been shown to influence prefrontal cortex (PFC) activation during both emotional processing and working memory (WM). Although COMT-genotype is not directly associated with MDD, it may affect MDD pathology by altering PFC activation, an endophenotype associated with both COMT and MDD. 125 participants, including healthy controls (HC, n=28) and MDD patients were genotyped for the COMT val158met polymorphism and underwent functional magnetic resonance imaging (fMRI-neuroimaging) during emotion processing (viewing of emotional facial expressions) and a WM task (visuospatial planning). Within HC, we observed a positive correlation between the number of met-alleles and right inferior frontal gyrus activation during emotional processing, whereas within patients the number of met-alleles was not correlated with PFC activation. During WM a negative correlation between the number of met-alleles and middle frontal gyrus activation was present in the total sample. In addition, during emotional processing there was an effect of genotype in a cluster including the amygdala and hippocampus. These results demonstrate that COMT genotype is associated with relevant endophenotypes for MDD. In addition, presence of MDD only interacts with genotype during emotional processing and not working memory.

## Introduction

Major depressive disorder (MDD) is a disorder characterized by abnormal interactions between cortical and subcortical structures [1,2] and altered catecholamine neurotransmission [3,4]. These disturbances affect both emotion processing [2] and executive functioning [5], and MDD is characterized by abnormal prefrontal activation during tasks tapping into these functions [2,5-12].

Catecholaminergic neurotransmission plays a central role in emotional and cognitive processing and it has recently been hypothesized that abnormal dopamine levels in the striatum contribute to altered cortical-subcortical interactions in MDD [2]. Catechol-O-methyltransferase (COMT) is an enzyme that breaks down catecholamines such as dopamine and norepinephrine, and is mainly present in prefrontal and temporal cortical areas [13,14]. A common polymorphism in the COMT-gene (i.e. rs4680, val158met; leading to an amino acid change of valine [val] to methionine [met]) results in altered COMT activity. Met-homozygotes have a three to four fold lower activity of COMT compared to val-homozygotes, with heterozygotes showing intermediate levels [15]. Consequentially, met-carriers have higher cortical concentrations of dopamine [16]. COMT-genotype variability has been postulated as an evolutionary switch toward a more cognitive versus a more emotional mental processing style [17].

In a meta-analysis investigating the effects of val158met genotype on prefrontal cortex (PFC) activation, it was shown that during emotional processing tasks the number of met-alleles correlated positively with PFC activation [18], located primarily in the inferior frontal gyrus (BA 45 and 47) [19-21]. This finding was interpreted as less efficient processing in met-carriers [18]. In contrast, during working memory tasks the number of met-alleles was negatively correlated with activation in the middle and superior frontal gyri (BA 9 and 46) [22-26] and IFG [27,28], implying less efficient processing in val-carriers in analogy with Mier et al. [18].

In addition to the PFC, during negative emotional processing, COMT-genotype has also shown to have an effect on amygdala activation. However, the results regarding the direction of the effect are inconsistent. Some studies reported a positive association between activation and the number of met-alleles [20,29-31], whereas others showed a negative [21,32,33] or an absent association [19]. Although the direction of the effect on the amygdala is not as clear as the effect on the PFC, there could be an influence of COMT genotype on amygdala functioning.

A direct association between MDD and val158met genotype has not been demonstrated [34]. We propose, however, that PFC and/or amygdala activation might be an endophenotype in studying the genetic basis of MDD. An endophenotype has been defined as a (neuro) biological substrate underlying a disease and to be more closely related to the effects of the gene [35]. Endophenotypes are often investigated in samples of healthy participants, but the presence of psychiatric disorders may modulate the effects of val158met genotype on brain activation [21,36]. To date, however, such modulatory effects of MDD on the association between val158met and regional brain activation has not been investigated.

The main aim of this study was to investigate the interaction between val158met genotype and depression using PFC and amygdala activation as endophenotype. For this purpose, we investigated whether the presence of MDD affected the opposing effects of genotype on PFC and amygdala activation during emotional and working memory tasks previously observed in healthy subjects. We measured brain activation with the use of fMRI-neuroimaging during a facial expression task and the “Tower of London” task, which is a visuospatial planning task known to activate a similar brain network as other working memory tasks [37]. Because of the variety in findings of PFC locations related to COMT-genotype [18], the entire lateral PFC was taken as our region of interest (ROI). The amygdala was used as an extra ROI in the analysis of the emotional processing task. In addition, we performed a whole-brain analysis to identify activations in other areas associated with val158met genotype.

## Materials and Methods

### Participants

Participants were selected from the multicenter Netherlands Study of Depression and Anxiety (NESDA [38]) which involved the University Medical Center Groningen (UMCG), VU University Medical Center, Amsterdam (VUmc) and Leiden University Medical Center (LUMC).

Exclusion criteria for all participants were presence or history of major internal and neurological disorder with potential central nervous system sequelae; current use of a beta-blocker; hypertension >180/130 mm Hg; age over 57 years; MRI incompatible implants or tattoos; use of psychotropic medication other than selective serotonin reuptake inhibitors (SSRIs) or infrequent use of benzodiazepines (oxazepam or diazepam, maximum of three times a week and not within 48 hours before scanning); and incomplete MRI data and/or overall task performance below 75% correct trials on the Tower of London task to maximize the likelihood of analyzing planning-related activation. A word encoding and recognition task [39] was also used as an exclusion criterion, to confirm task engagement due to the two-choice answer method.

Genotype data were obtained in addition to functional MRI data during emotional and working memory processing from 125 participants. Of these, 97 had experienced major depressive disorder (MDD) during their life as established using the Composite International Diagnostic Interview (CIDI) [40], and were therefore considered to have the phenotype depression vulnerability whereas the other 28 were healthy controls (Table 1 and Table 2). Presence of anxiety diagnosis was allowed because of the high comorbidity with MDD (n=66), presence of any other psychiatric disorder was an exclusion criteria in the overall NESDA study.

**Table 1:** Demographic and clinical details divided by genotype.

						**met/met**	**met/val**	**val/val**	**test**	***p***
N				31			65	29		
Gender		#females (%)		22 (71.0)			38 (58.5)	19 (65.5)	χ^2^(2)=1.50	.47
Age		mean (SD)		34.19 (10.03)			38.43 (9.73)	37.62 (9.77)	F(2,122)=1.99	.14
Education (in years)		mean (SD)		13.32 (2.62)			12.66 (3.41)	12.21 (3.08)	F(2,122)=0.96	.39
Center		UMCG/AMC/LUMC*		11/7/13			27/10/28	8/10/11	χ^2^(4)=4.61	.33
Diagnosis		HC/MDD		11/20			10/55	7/22	χ^2^(2)=4.94	.08
MADRS		mean (SD)		8.29 (7.71)			13.05 (10.49)	12.21 (10.49)	F(2,122)=2.49	.09
BAI		mean (SD)		9.16 (7.86)			11.38 (10.70)	12.10 (11.45)	F(2,122)=0.71	.49
SSRI use		N (%)		6 (19.4)			16 (24.6)	10 (34.5)	χ^2^(2)=1.87	.39
*Faces task - reaction times (milliseconds*)										
angry				800.83 (116.37)			846.57 (177.81)	807.31 (186.80)		
fear				840.25 (117.49)			884.41 (193.82)	844.61 (186.77)		
happy				854.26 (105.74)			890.12 (180.94)	875.90 (178.41)		
neutral				866.37 (117/40)			891.65 (173.81)	844.63 (178.52)		
sad				833.67 (105.04)			878.21 (167.88)	854.30 (191.35)		
*Tower of London task - reaction times (seconds*)										
step 1					4.56 (1.38)		4.81 (1.54)	4.91 (1.23)		
step 2					5.95 (2.06)		6.00 (1.79)	6.13 (1.50)		
step 3					8.30 (3.82)		7.98 (2.45)	8.07 (2.00)		
step 4					11.72 (4.91)		11.40 (4.00)	11.80 (3.10)		
step 5					15.43 (5.95)		16.14 (5.15)	17.00 (6.34)		
*Tower of London task - accuracy (proportions*)										
step 1					0.96 (0.05)		0.97 (0.05)	0.96 (0.08)		
step 2					0.94 (0.07)		0.93 (0.10)	0.90 (0.09)		
step 3					0.94 (0.08)		0.92 (0.11)	0.93 (0.09)		
step 4					0.87 (0.17)		0.81 (0.16)	0.81 (0.19)		
step 5					0.82 (0.21)		0.76 (0.20)	0.80 (0.16)		

#for these variables was corrected in the main analyses. * UMCG: University Medical Center Groningen; AMC: Amsterdam Medical Center; LUMC: Leiden University Medical Center.

**Table 2:** Demographic and clinical details partitioned according to diagnoses.

		**HC**	**patients**	**test**	***p***
N		28	97		
Genotype	mm/mv/vv	11/10/7	20/55/22	χ^2^(2)=4.94	.08
Gender	#females (%)	14 (50%)	65 (67%)	χ^2^(1)=2.70	.10
Age	mean (SD)	40.93 (8.67)	36.11 (9.99)	t(123)=2.31	0.02#
Center	UMCG/AMC/LUMC*	8/10/10	38/17/42	χ^2^(2)=4.29	.12
Education (in years)	mean (SD)	14.79 (2.50)	12.12 (3.08)	t(123)=4.19	<.001#
MADRS	mean (SD)	1.25 (1.99)	14.68 (9.33)	t(123)=7.55	<.001
BAI	mean (SD)	2.14 (2.58)	13.56 (10.20)	t(123)=5.85	<.001
SSRI use	N (%)	-	32 (33)		
*Faces task - reaction times (milliseconds*)					
angry		813.98 (166.37)	829.62 (168.06)		
fear		865.36 (168.11)	863.90 (179.33)		
happy		878.66 (151.62)	877.72 (168.58)		
neutral		878.16 (138.80)	873.41 (169.73)		
sad		852.12 (154.50)	864.36 (163.17)		
*Tower of London task - reaction times (seconds*)					
step 1		4.51 (1.19)	4.85 (1.48)		
step 2		5.79 (1.62)	6.08 (1.84)		
step 3		8.35 (3.54)	8.00 (2.49)		
step 4		12.33 (4.85)	11.35 (3.77)		
step 5		16.53 (4.52)	16.06 (5.92)		
*Tower of London task - accuracy (proportions*)					
step 1		0.96 (0.07)	0.96 (0.05)		
step 2		0.93 (0.08)	0.93 (0.09)		
step 3		0.93 (0.09)	0.93 (0.09)		
step 4		0.84 (0.15)	0.82 (0.17)		
step 5		0.81 (0.14)	0.77 (0.21)		

#for these variables was corrected in the main analyses. * UMCG: University Medical Center Groningen; AMC: Amsterdam Medical Center; LUMC: Leiden University Medical Center.

### Ethic statement

This study was approved by the Ethical Committees at the University Medical Center Groningen, VU University Medical Center, Academic Medical Center, Amsterdam, and the Leiden University Medical Center. All participants provided written informed consent. The study was conducted in accordance with the declaration of Helsinki.

### Clinical measurements

Depression severity was determined by the Montgomery-Åsberg rating scale (MADRS) [41] and anxiety severity was determined by the Beck Anxiety Inventory (BAI) [42].

### Genotyping

Genotyping was performed in the context of the genome wide association (GWA) study of the Genetic Association Information Network (GAIN), its method having been described in detail elsewhere [43]. Perlegen Sciences (Mountain View, CA, USA) performed all genotyping according to standard operating procedures. High-density oligonucleotide arrays were used yielding 599,164 single nucleotide polymorphisms (SNP). These arrays included the val158met SNP (rs4680). In this sample, the genotype distribution of the rs4680 did not differ significantly from the expected numbers calculated on the basis of observed allele frequencies according to the Hardy-Weinberg equilibrium (HWE, χ^2^_(1)_=0.2, *p*>.65).

### MRI protocol

#### Emotion processing

The paradigm used in this study was described before [44]. Briefly, participants viewed photographs from a widely used set of emotional facial expressions [45] (angry, fearful, happy, neutral and sad) and were requested to make gender judgments. Twenty-four stimuli were selected for each of five facial expressions, comprising 12 female and 12 male faces. Each face was not presented more than four times. As control condition, a scrambled face with an arrow (“<<” or “>>”) was shown indicating which button to press. The control condition (scrambled faces) was presented 80 times. The pictures were shown for 2.5 seconds. Responses and reaction times were recorded.

#### Working memory

The Tower of London (ToL) task was used to measure working memory [11,46]. On the screen two pictures were shown with colored balls on rods, representing two configurations, one start and one goal. In the task condition, participants had to work out the number of steps (ranging from one to five) needed to reach the target configuration. In the control condition, they were instructed to count the number of blue and yellow balls. We used a pseudorandomized, self-paced design with maximal response duration of 60 seconds for each trial. Responses and reaction times were recorded.

### Image acquisition

All participants were scanned using a Philips 3T MR-scanner at the three different sites. A SENSE-8 -channel head coil was used for radio frequency transmission and reception in Groningen and Leiden. In Amsterdam a SENSE-6 channel head coil was used.

For every participant, echo planar images (EPI) were obtained, entailing a T2*weighted gradient echo sequence using axial whole brain acquisition, with an interleaved slice acquisition order and the following settings: repetition time (TR) = 2300 ms, echo time (TE) = 28.0 at UMCG and 30 at AMC and LUMC, and a flip angle of 90°. At UMCG 39 slices per EPI volume were acquired, with a matrix size of 64x64 voxels and an in-plane resolution of 3x3 mm. At AMC and LUMC 35 slices per EPI volume were acquired, with a matrix size of 96x96 voxels and an in-plane resolution of 2.29x2.29 mm. The slices had a 0 mm gap and 3 mm thickness. The images were acquired parallel to the anterior-posterior commissure plane. In addition, a T1-weighted anatomical MRI was made (TR = 9 ms, TE = 3.5 ms, matrix size 256x256, voxel size: 1x1x1 mm).

### Data analyses

#### Demographic, clinical and behavioral data

For the analysis of clinical and behavioral data SPSS version 16.0 was used. To test for genotype and presence of psychopathology effects on demographic data, Chi-square test or analysis of variance was used, whenever appropriate. To investigate an association between genotype and depression and anxiety symptoms, a multivariate analysis of variance (MANOVA) was performed with genotype as independent variable and MADRS and BAI-scores as dependent variables.

For behavioral data, repeated measures ANOVAs were used for reaction times (faces task and TOL) and accuracy (TOL) with emotional expression as a within-subject factor and presence of psychopathology and number of met-alleles as between-subject factors.

#### Preprocessing of functional data

Functional imaging data were preprocessed and analyzed using Statistical Parametric Mapping software (SPM5; http://www.fil.ion.ucl.ac.uk/spm/) implemented in Matlab 7.1.0 [47]. Preprocessing included slice time correction, image realignment, registration of the T1-scan to the mean EPI, warping to MNI-space as defined by the SPM5 T1-template, reslicing to 3×3×3 mm voxels and spatial smoothing using an 8-mm FWHM Gaussian kernel. Movement of the participant of >3 mm in any direction resulted in exclusion of all data from further analysis.

#### First-level analyses

For the emotion processing task, for every participant, hemodynamic responses for each stimulus were modeled, including regressors for each condition (angry, fearful, happy, neutral and sad) and for baseline trials (scrambled faces). Low frequency noise was removed by applying a high pass filter of 128 s. For each participant, contrast images were produced for “negative vs. scrambled”, consisting of the expressions angry, fear and sad and “positive vs. scrambled”, including happy facial expressions.

For the working memory task, for every participant, hemodynamic responses for each stimulus were modeled. The model included regressors for each number of steps and for baseline items. Again, low frequency noise was removed by applying a high pass filter of 128 s. For each participant, contrast images for “task load” [with trial types 1–5 having weights (-1.5, -1, -0.5, 1, 2)] were calculated.

#### Second level analyses

We performed separate factorial models for each first-level contrast (i.e. negative faces vs. scrambled, positive faces vs. scrambled, and ‘task load’ (ToL)). Diagnosis was entered as factor. Comparable to previous studies (e.g. [20,22,48]), gene-dose of COMT genotype was entered as regressor, coded as the number of met-alleles (0, 1 or 2). This regressor was modeled in interaction with diagnosis. To control for potential confounding effects of gender on genotype, we added gender, center (two dummy variables), age, education (last two adjusted for the group mean) as covariates. A small volume correction was used for our regions of interest (ROIs). Based on the literature (see introduction), we chose the entire left and right lateral PFC (based on AAL library implemented in WFU pickatlas: superior frontal gyrus, middle frontal gyrus, inferior frontal gyrus, medial frontal gyrus, see also Figure S1) as our ROI (left and right separately). In addition, for the emotional processing task we also included the bilateral amygdala as ROI.

We tested for correlations between genotype and PFC activation independent of diagnosis and differences in these correlations between HC and patients, all within one model. For the correlation between number of met-alleles and brain activation, a threshold was set at *p*<.05 family wise error (FWE) cluster-level corrected for the spatial extent of the search volume of our ROIs. The interaction between genotype and diagnosis was inspected with an F-test at a threshold of *p*<.001. The *post-hoc* t-tests had to meet *p*<.05 FWE cluster-level corrected for the spatial extent of the search volume of our ROIs. For completeness, we also report whole-brain analyses at a threshold of *p*<.05 family wise error (FWE) whole-brain cluster-level corrected.

We extracted the mean activation of the clusters with the use of MarsBaR [49] as a measure of the strength of the brain activation per participant to visualize the data in a scatterplot.

## Results

### Demographic and Clinical data

Genotype was not related to gender (χ^2^(2)= 1.50, *p*=.47), age (F_(2, 122)_=1.99, *p*=.14) or education (F_(2, 122)_=0.96, *p*=.34, Table 1). The multivariate ANOVA did not reveal an association between genotype and MADRS and/or BAI-scores in the total sample (Pillai’s trace F_(4, 244)_=1.41, *p*=.23) or in patients only (Pillai’s trace F_(4, 188)_=0.60, *p*=.66). There was also no significant association between genotype and diagnosis (χ^2^(6)=7.12, *p*=.31) or genotype and SSRI-use (χ^2^(2)= 1.87, *p*=.39, Table 1).

### Emotional processing task

#### Behavioral responses

A main effect of emotional expression was present on reaction times (F_(4, 114)_=5.55, *p*<.001): all participants were faster in responding to angry faces than to other emotional expressions. The number of met-alleles or presence of psychopathology or interactions between these variables did not affect RTs, also not in interaction with emotional expression (all F<1.06, all *p*>.37).

#### Neural responses

**Figure 1:**
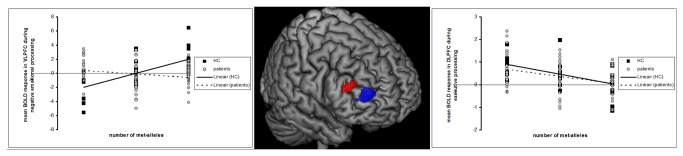
Prefrontal cortical activity correlated with number of met-alleles during emotional and working memory processing: hypothesis testing. Red represents a positive correlation between number of met-alleles during processing of negative emotional facial expressions only in HC. The graph depicting the correlation between activity and number of met-alleles is located on the left. Blue represents a negative correlation between number of met-alleles and activity during working memory over all subjects. The graph depicting the correlation between activity and number of met-alleles is located on the right. The threshold was set at p<.001 uncorrected and the peak voxel had to survive *p*<.05 family wise error (FWE) corrected for the spatial extent of the PFC with a small volume correction (see methods).

There was no significant correlation between genotype and activation during processing of negative emotional expressions in the PFC or amygdala in the overall sample. However, we observed an interaction between genotype and presence of MDD (Brodmann area [BA] 45, [x=39 y=39z=18], F(1, 116)=24.74), reflecting a positive correlation between the number of met-alleles and activation in the right inferior frontal gyrus (IFG) in HC (BA45, Figure 1, Z=4.49, k=35, *p*_FWE_=.014), which was absent in patients. There were no significant main effects for diagnosis. However, a trend was seen of more activation for MDD patients than HC in the same location as the interaction effect was present ([x=39 y=39z=18], Z=4.12, k=8, *p*_FWE_=.19, *p*<.001 uncorrected).

**Figure 2:**
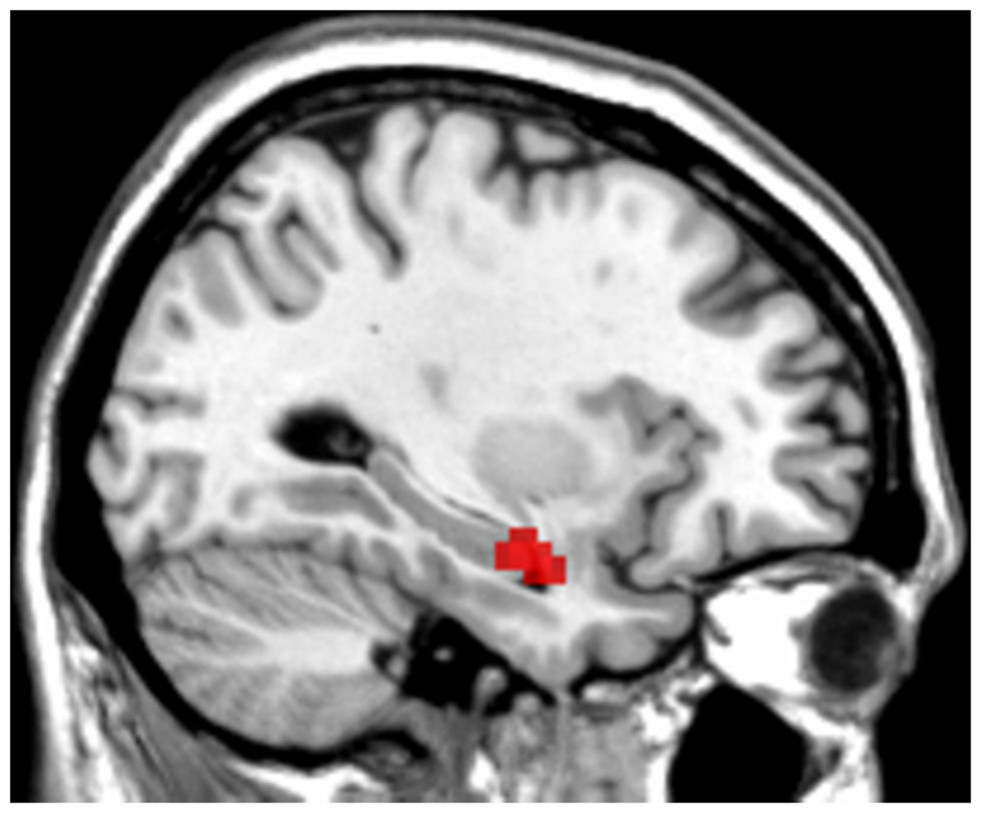
Hippocampal activation during emotional processing showing correlations with number of met-alleles: exploratory analysis. A. Positive correlation between the number of met-alleles and activitation in the left hippocampus/amygdala. A threshold was set at p<.001 uncorrected.

During processing of positive emotional expressions, there was no significant correlation between genotype and PFC activation, also not in interaction with diagnosis. In the left amygdala, there was a positive correlation between number of met-alleles and activation in the overall sample ([x=-27 y=-6z=-18], k=2, Z=3.38, p_FWE_=.036) However, our whole-brain analysis showed that this activated cluster was mostly located in the hippocampus (Figure 2, [x=-33 y=-3z=-21], Z=4.06, k=19, *p*_FWE_=.38, whole brain corrected, p<.001 uncorrected). There were no significant main effects for diagnosis.

After excluding patients using SSRIs the results in the PFC were highly similar. There were no significant correlations between number of met-alleles and brain activation in response to the contrasts “negative vs. neutral expressions” or “positive vs. neutral expressions”.

### Working memory task

#### Behavioral responses

With increasing difficulty of the task (increase in number of steps), reaction times (RTs) became longer (F(1.71, 203.92)=271.81, *p*<.001) and accuracy decreased (F(2.68, 318.74)=34.50, *p*<.001). There were no main effects of number of met-alleles or presence of psychopathology on RT or on accuracy nor in interaction with each other (all F<1.21, *p*>.30) or in interaction with the number of steps (all F<0.91, *p*>.40).

#### Neural responses

Over the whole sample, there was a significant negative association between number of met-alleles and right middle frontal gyrus (MFG, BA46/10) activation (Figure 1, [x=33 y=48z=12], k= 34, Z=3.82, *p*_*FWE*_=.024). The correlation between genotype and PFC activation did not reach significance within HC (Z=3.16, *p*_*FWE*_=.39, *p*_uncorr_=.001) or MDD (Z=3.53, *p*_*FWE*_=.08, *p*_uncorr_<.001) separately. There was also no interaction between diagnosis and genotype on PFC activation. In addition, no other areas emerged as significantly from the whole-brain analysis. Excluding medicated patients did not change these results.

## Discussion

The aim of the current study was to investigate the influence of the val158met polymorphism in the gene coding for catechol-O-methyltransferase (COMT) on cortical functioning, and whether the presence of major depressive disorder (MDD) moderated these associations. Met-homozygotes have been associated with lower enzymatic activity of COMT compared to val-homozygotes [15], with consequently higher cortical dopamine concentrations [16]. We showed that during emotional processing there was an interaction between diagnosis and COMT-genotype: in healthy participants the number of met-alleles was associated with higher activation in the inferior frontal gyrus (IFG), whereas in MDD patients IFG activation was not affected by genotype. In addition, during working memory, carrying the met-allele was associated with lower activation in the middle frontal gyrus (MFG) in both HC and MDD patients. Thus, our results show a more extensive moderating effect of MDD on the relation between COMT genotype and PFC activation during emotional processing than during working memory.

A moderating effect was present of psychopathological status on the effect of val158met genotype on IFG (BA45) activation during processing of negative facial expressions. Within healthy participants the number of met-alleles was positively correlated with activation in the IFG, whereas within patients, there was no effect of val158met genotype in IFG activation. The IFG has been associated with emotional processing [50] and behavioral inhibition [51,52]. In addition, greater activation in this area has also been associated with inhibition of negative emotions [6,50,53]. It has been suggested that met/met-carriers show impairments in emotion paradigms in combination with more PFC activation, reflecting less efficient cortical functioning [18]. This might be associated with less efficient inhibition of emotional distraction due to negative facial expressions. In our sample, healthy met/met-genotype carriers showed more activation in the IFG, but did not show any difference in behavioral responses. Therefore, hyperactivation of the IFG might represent a compensation mechanism to reach the same level of functioning. Within patients, there was no effect of val158met genotype in IFG activation; the met/met-carriers showed a similar response as val/val-carriers, but in general lower than HC. It could be suggested that the effects of depression on brain activation during emotional processing are greater than the effects of COMT-genotype, obscuring any compensatory activation in depressed met/met-carriers as was found in healthy participants.

Depression is primarily a disorder of emotion [54]. Therefore, it is an interesting finding that the interaction with psychopathology was only present during emotional processing. During working memory, there was less activation in the MFG related to the met-allele in both patients and healthy controls. This was accompanied by an absence of performance differences, indicating that WM processing was intact. The task was probably not too easy, given the increase in reaction times and decrease in accuracy with increasing planning load. The direction of the effect of val158met genotype on brain activation is in line with the meta-analysis of Mier et al. (2010) and suggests a compensation in cortical processing in val/val-carriers during working memory. In addition, the absent of an interaction with psychopathology, is in agreement with other studies showing normal planning performance in MDD outpatients, suggesting relatively unaffected cognitive functioning [11,55-57]. Notably, impairments in working memory are particularly present in severely depressed patients (reviewed by [58,59]), whereas emotional processing is thought to be disturbed already before the onset of the first depressive episode (reviewed by [60,61]). This could explain that psychopathological status of our relatively mild depressed outpatient sample moderated the effect of genotype only during emotional processing and not during working memory.

In addition to the prefrontal cortex, during processing of positive emotional facial expressions, there was a positive correlation between the number of met-alleles and activitation in the amygdala. However, these voxels were part of a larger cluster, which was mostly located in the hippocampus. Therefore, the effect in the amygdala likely represents a partial volume effect from the hippocampal activation. Besides the cortex, COMT is also strongly expressed in the hippocampal formation, especially the dentate gyrus [13]. Indeed, there are previous reports of positive correlations between number of met-alleles and hippocampal activity during memory processing [62-64] and also one report of a positive correlation between the number of met-alleles and activitation in the left hippocampus during processing of unpleasant stimuli [20]. However, to our knowledge, our results show for the first time an association between COMT-genotype and activation in the hippocampus in response to positive emotional stimuli. Although the hippocampus has primarily been associated with memory processing, it has also been associated with inhibition of stress responses (e.g. [65]) and emotion processing [66]. As described above, it has been suggested that met/met-carriers are less emotionally stable [17]. This might lead to a compensation in brain activation in a broader network of emotion processing related brain areas, including the prefrontal cortex [18] and subcortical areas.

A limitation of this study was the relatively small sample size, which does not allow for strong conclusions on genetic associations. In addition, there were many variables in our sample that could have been confounding factors (e.g. medication use, scanner site). For all effects, we have tested for a possible influence of these confounding factors. Despite not finding any influence of these possible confounders, such effects cannot be fully discarded due to our limited sample size. Not with standing, we think our findings are of interest as this is the first study that replicates the meta-analysis results of Mier et al. and their hypothesis of current literature on pleiotropic effects of val158met genotype on PFC activation in one single sample. In addition, this is to our knowledge one of the first studies to suggest that this effect is moderated by psychopathology.

To conclude, these results showed that the influence of COMT val158met genotype on prefrontal function is different in depressed patients compared to HC during emotional processing, but not during WM. This SNP thus appears to play a role in the etiology or expression of MDD or both with brain activation as a promising endophenotype. In addition, our study revealed that COMT-genotype influenced activation during emotional processing in subcortico-limbic areas (VTA and hippocampus). It could be speculated that increased activation in these areas in met-carriers may contribute to less emotional resilience and render carriers more vulnerable to affective disorders.

## Supporting Information

Figure S1 **Definition of regions of interest.** The left (red) and right (blue) prefrontal cortex were selected as our regions of interest based on the automatic atlas library regions: superior frontal gyrus, middle frontal gyrus, inferior frontal gyrus, medial frontal gyrus. The orbital part of the PFC was omitted due to scanner artefacts.
(DOCX)
