# Supplementary material for: Influence of COMT val158met Genotype on the Depressed Brain during Emotional Processing and Working Memory
Source: PLoS One. 2013 Sep 12;8(9):e73290. doi: 10.1371/journal.pone.0073290 (PMC3772077; doi:10.1371/journal.pone.0073290)

**Supplementary information**

Figure S1: Definition of regions of interest

The left (red) and right (blue) prefrontal cortex were selected as our regions of interest based on the automatic atlas library regions: superior frontal gyrus, middle frontal gyrus, inferior frontal gyrus, medial frontal gyrus. The orbital part of the PFC was omitted due to scanner artefacts.


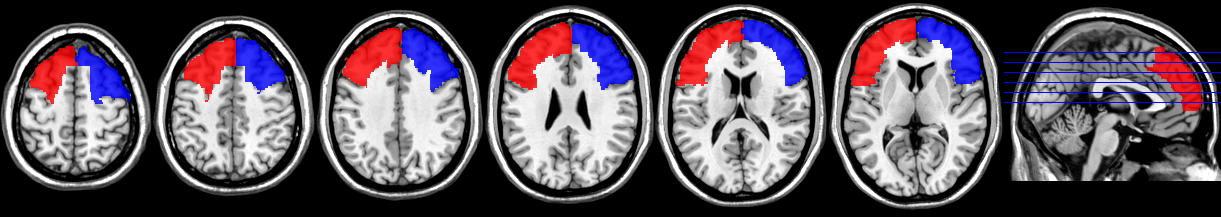

Supplement: Figure S1 — Definition of regions of interest. The left (red) and right (blue) prefrontal cortex were selected as our regions of interest based on the automatic atlas library regions: superior frontal gyrus, middle frontal gyrus, inferior frontal gyrus, medial frontal gyrus. The orbital part of the PFC was omitted due to scanner artefacts. (DOCX) [file pone.0073290.s001.docx]
